# Assessing SARS-CoV-2-specific T-cell reactivity in late convalescents and vaccinees: Comparison and combination of QuantiFERON and activation-induced marker assays, and relation with antibody status

**DOI:** 10.1371/journal.pone.0285728

**Published:** 2023-05-23

**Authors:** Arianna Gatti, Gaetano Zizzo, Massimo De Paschale, Antonio Tamburello, Laura Castelnovo, Paola Maria Faggioli, Pierangelo Clerici, Bruno Brando, Antonino Mazzone

**Affiliations:** 1 Laboratory of Haematology, Transfusion Center, Legnano Hospital, ASST Ovest Milanese, via Papa Giovanni Paolo II, Legnano, Milan, Italy; 2 Department of Internal Medicine, Legnano and Cuggiono Hospitals, ASST Ovest Milanese, via Papa Giovanni Paolo II, Legnano, Milan, Italy; 3 Unit of Microbiology, Legnano Hospital, ASST Ovest Milanese, via Papa Giovanni Paolo II, Legnano, Milan, Italy; University of Bologna / Romagna Local Health Authority, ITALY

## Abstract

**Objectives:**

Monitoring of SARS-CoV-2 spread and vaccination strategies have relied on antibody (Ab) status as a correlate of protection. We used QuantiFERON™ (QFN) and Activation-Induced Marker (AIM) assays to measure memory T-cell reactivity in unvaccinated individuals with prior documented symptomatic infection (late convalescents) and fully vaccinated asymptomatic donors (vaccinees).

**Methods:**

Twenty-two convalescents and 13 vaccinees were enrolled. Serum anti-SARS-CoV-2 S1 and N Abs were measured using chemiluminescent immunoassays. QFN was performed following instructions and interferon-gamma (IFN-γ) measured by ELISA. AIM was performed on aliquots of antigen-stimulated samples from QFN tubes. SARS-CoV-2-specific memory CD4^+^CD25^+^CD134^+^, CD4^+^CD69^+^CD137^+^ and CD8^+^CD69^+^CD137^+^ T-cell frequencies were measured by flow cytometry.

**Results:**

In convalescents, substantial agreement was observed between QFN and AIM assays. IFN-γ concentrations and AIM^+^ (CD69^+^CD137^+^) CD4^+^ T-cell frequencies correlated with each other, with Ab levels and AIM^+^ CD8^+^ T-cell frequencies, whereas AIM^+^ (CD25^+^CD134^+^) CD4^+^ T-cell frequencies correlated with age. AIM^+^ CD4^+^ T-cell frequencies increased with time since infection, whereas AIM^+^ CD8^+^ T-cell expansion was greater after recent reinfection. QFN-reactivity and anti-S1 titers were lower, whereas anti-N titers were higher, and no statistical difference in AIM-reactivity and Ab positivity emerged compared to vaccinees.

**Conclusions:**

Albeit on a limited sample size, we confirm that coordinated, cellular and humoral responses are detectable in convalescents up to 2 years after prior infection. Combining QFN with AIM may enhance detection of naturally acquired memory responses and help stratify virus-exposed individuals in T helper 1-type (T_H_1)-reactive (QFN^pos^ AIM^pos^ Abs^high^), non-T_H_1-reactive (QFN^neg^ AIM^pos^ Abs^high/low^), and pauci-reactive (QFN^neg^ AIM^neg^ Abs^low^).

## Introduction

The COVID-19 pandemic, due to the SARS-CoV-2 coronavirus, is to date the greatest global health challenge of the 21st century. A full comprehension of the adaptive immune response to SARS-CoV-2 would enable more efficient public health policies to safeguard the most fragile and at risk groups. Monitoring of healthcare workers and general population, as well as effectiveness of vaccines, has so far relied mainly on serological antibody tests as correlates of protection [1]. However, long-lasting adaptive immunity is orchestrated by T cells [2, 3]. In particular, through the production of interferon-gamma (IFN-γ) and cell-to-cell interactions, effector memory CD4^+^ T helper 1 (T_H_1) and central memory follicular T helper (T_FH_) cells drive and mantain the protective antiviral response against SARS-CoV-2, both at the intracellular and extracellular level: on one side, by stimulating CD8^+^ cytotoxic lymphocytes to recognize and eliminate virus-infected cells; on the other side, by stimulating production of neutralizing antibodies with high affinity by germinal center B cells [4, 5]. While the antibody response is important to prevent virus entry into cells and the onset of clinical symptoms, early cellular response is then crucial to ensure rapid viral clearance and avoid disease progression towards lethal forms [2].

Immunization induced by natural infection encompasses the presence of virus-specific IgA antibodies in the airways as well as virus-reactive T cells against antigenic peptides derived not only from the Spike protein (S), but also from nucleocapsid (N), membrane, and nonstructural proteins, which are presented by professional antigen-presenting cells (MHC-II-restricted epitopes) or infected cells (MHC-I-restricted epitopes) [6–8]. Current vaccines, instead, substantially elicit S-specific T-cell clones and IgG antibodies [8, 9].

During the first wave of the pandemic in Italy, in which the ancestral (Alpha) strain was still dominant, we reported that patients hospitalized with COVID-19 were protected from rehospitalization at 3 months after discharge [10] and that subjects exposed to SARS-CoV-2 were protected from reinfection up to 12 months after prior infection [11], thereby suggesting a long-lasting protection exerted by naturally acquired immunity. Similar observations at 6 months were done among healthworkers in the UK [1]. Flourishing of new variants of concern (VOCs) highly mutated in the Spike protein (such as Omicron) has implied that the antibody response, induced by prior infection or vaccination, is often escaped, with frequent primary infections or reinfections and possible clinical symptoms or relapses, so far mostly mild. The wide repertoire of memory CD4^+^ and CD8^+^ T cells against S and non-S (mainly N) epitopes is, in fact, still preserved and able to protect from severe disease [6, 12, 13].

Therefore, growing interest is turning to virus-specific memory T-cell responses, in the aim to predict the actual immunity of each individual, on which to base more appropriate vaccination strategies in the future. T-cell analysis has mostly been focused on their ability to produce and release IFN-γ upon stimulation with SARS-CoV-2-derived peptides, using the enzyme-linked immunosorbent ELISpot or IFN-γ release assays (IGRA), such as QuantiFERON (QFN), or the IFN-γ intracellular staining (ICS) measured by flow cytometry. Another way to detect T-cell reactivity is studying by flow cytometry the T-cell upregulation of activation-induced markers (AIM) following viral antigen exposure [3]. This method would allow a more comprehensive assessment of SARS-CoV-2-specific T-cell responses, since outcomes are independent of T-cell commitment and cytokine response [14].

So far, these techniques have mainly been used in COVID-19 to examine T-cell function in recently infected patients [7, 15] or to estimate cellular immunity induced by mRNA vaccines [12, 16–19]. In the present study, we compared and combined QFN and AIM assays, and analyzed memory CD4^+^ and CD8^+^ T-cell responses along with anti-S1 and anti-N antibody responses, in order to more exhaustively evaluate the immunological memory persisting in immunocompetent unvaccinated individuals with prior symptomatic infection documented up to 2 years earlier (late convalescents), compared to fully vaccinated asymptomatic donors (vaccinees).

## Materials and methods

### Subjects

Peripheral blood samples were collected between January and June 2022. Overall, 22 consecutive individuals with prior symptomatic SARS-CoV-2 infection and no vaccinations (unvaccinated cases) and 13 donors with no prior symptomatic infection and receiving three doses of mRNA COVID-19 vaccines (vaccinated controls) were enrolled. Unvaccinated cases (7 M, 15 F) had a median age of 52.5 years old and a median time since primary infection (documented by RT-PCR on nasopharyngeal swabs) of 476 days (swab positivity from 4 months to 2 years earlier). Most had had a mild form of COVID-19, two had developed moderate-to-severe disease (bilateral pneumonia), and one had experienced critical disease (intensive care unit admission and invasive mechanical ventilation). Vaccinated controls (4 M, 9 F) had a median age of 51 years old and a median time since complete vaccination of 147.5 days (booster dose at least 4 months earlier).

The study was conducted in accordance with the ethical principles reported in the Declaration of Helsinki and approved by the local Ethics Committee (Milano Area 3, Niguarda Hospital); all subjects gave their written informed consent to participate in the study.

### Serological immunoassays

Participants were evaluated for the presence of circulating anti-SARS-CoV-2 Spike protein subunit 1 (anti-S1) antibodies (total and IgG) (VITROS®, Ortho-Clinical Diagnostics, Rochester, US) and anti-SARS-CoV-2 Nucleocapsid protein (anti-N) antibodies (Roche, Mannheim, Germany), by means of chemiluminescent immunoassays (CLIA).

### QuantiFERON™ SARS-CoV-2 assay

The QuantiFERON™ SARS-CoV-2 assay (QFN) (Qiagen, Hilden, Germany) was performed following the manufacturer’s instructions. The assay included four blood sample collection tubes: Nil tube, as negative control for background subtraction (e.g., nonspecific IFN-γ level in the blood); Ag1 tube, for stimulation of S1-specific CD4^+^ T cells; Ag2 tube, for stimulation of S1- and S2-specific CD4^+^ and CD8^+^ T cells; and Mitogen tube, as positive control. After incubation and centrifugation, the amounts of IFN-γ released in supernatants were measured using an enzyme-linked immunosorbent assay (ELISA), and results were expressed in international units per milliliter (IU/mL). Nil values were subtracted from Ag1 and Ag2 values to obtain final adjusted IFN-γ concentrations (thus possibly including negative adjusted values). A QFN-reactive status was defined when, in Ag1 and/or Ag2 tubes, an IFN-γ concentration of at least 0.15 IU/ml was detectable.

### Activation-induced marker assay

**Fig 1:**
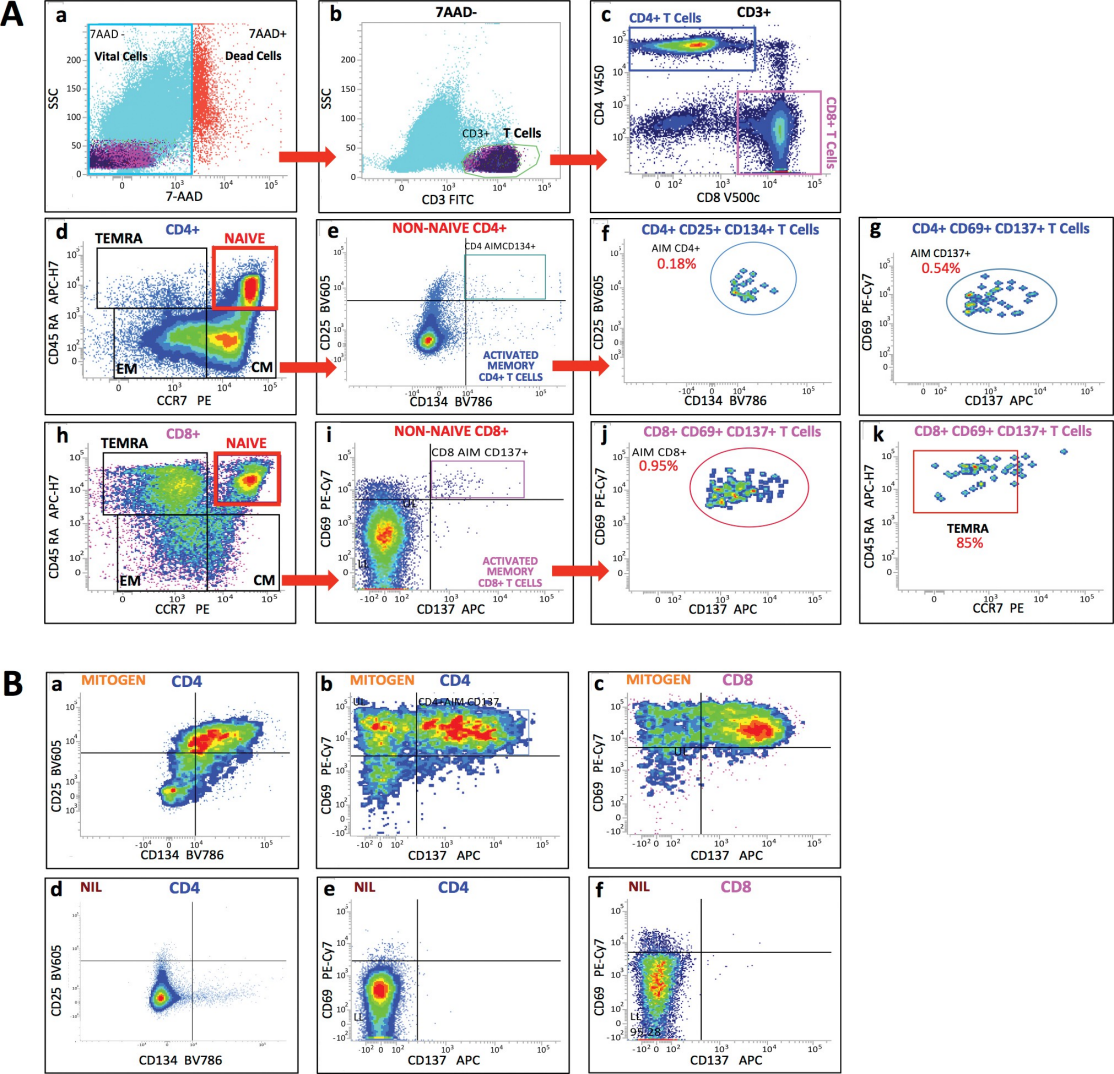
Gating strategies for detection of AIM^+^ CD4^+^ and AIM^+^ CD8^+^ T cells by flow cytometry, and positive and negative controls. ***A)*** Nonviable cells were excluded from analysis using 7-amino-actinomycin D (7-AAD) (**a**); T cells were identified using CD3 (**b**); CD4^+^ T cells were distinguished from CD8^+^ T cells (**c**); CD4^+^ (**d**) and CD8^+^ (**h**) T cells were divided, based on CD45RA and CCR7 expression, into: naïve (CCR7^+^ CD45RA^+^), central memory (T_CM_, CCR7^+^ CD45RA^-^), effector memory (T_EM_, CCR7^-^ CD45RA^-^), and effector memory CD45RA^+^ T cells (T_EMRA_, CCR7^-^ CD45RA^+^); virus-reactive memory (non-naïve) AIM^+^ CD4^+^ T cells were identified by the co-expression of CD25 and CD134 (**e, f**) or CD69 and CD137 (**g**); virus-reactive memory (non-naïve) AIM^+^ CD8^+^ T cells were identified by the co-expression of CD69 and CD137 (**i, j**); AIM^+^ (CD69^+^ CD137^+^) CD8^+^ T cells predominantly showed the T_EMRA_ phenotype (CCR7^-^ CD45RA^+^) (**k**). ***B)*** Samples from Mitogen (**a, b, c**) and Nil (**d, e, f**) tubes were used as positive and negative controls, respectively, to define thresholds for AIM^+^ (CD25^+^ CD134^+^) CD4^+^ T cells (**a, d**) and AIM^+^ (CD69^+^ CD137^+^) CD4^+^ (**b, e**) or CD8^+^ T cells (**c, f**).

An in-house activation-induced marker (AIM) assay was carried out on 200 μL aliquots of blood samples incubated in the QFN tubes, which were aspirated at the end of incubation and before centrifugation. Such procedure did not interfere with the QFN assay and allowed to quantify and characterize the phenotype of SARS-CoV-2-reactive T-cell subsets exposed to antigen peptides (Ag1 and Ag2) in QFN tubes and therein activated at the single-cell level. Blood samples were stained with a stain-lyse-wash technique, using pre-titrated, fluorochrome-conjugated antibodies recognizing: CD3-FITC, CD4-V450, CD8-V500c, CD45RA-APC-H7, CCR7-PE, CD25-BV605, CD134-BV786, CD69-PE-Cy7, and CD137-APC (Becton Dickinson). 7-amino-actinomycin D (7-AAD) was used to rule out nonviable cells in the analysis. Readings were performed on a 12-color FACSLyric flow cytometer (Becton Dickinson) equipped with three lasers and FACSuite software. A minimum of 30,000 CD3^+^ T-cell events was acquired in all cases. To define and quantitate each cell subset, a cluster of at least 20 homogeneous events was required to ensure a lowest level of quantitation of 0.06%, as recommended for flow cytometric analysis of rare events. Naïve CD4^+^ and CD8^+^ T cells, defined by the co-expression of CD45RA and CCR7, were excluded from analysis. AIM expression was assessed on the remaining population, representing memory T cells, which were further divided into central memory (T_CM_, CCR7^+^CD45RA^-^), effector memory (T_EM_, CCR7^-^CD45RA^-^), and effector memory CD45RA^+^ T cells (T_EMRA_, CCR7^-^CD45RA^+^) (**Fig 1A**). AIM^+^CD4^+^ T cells were defined by the co-expression of CD25 and CD134 (OX40), or CD69 and CD137 (4-1BB), while AIM^+^CD8^+^ T cells were defined by the co-expression of CD69 and CD137. The percentages of AIM^+^CD4^+^ or AIM^+^CD8^+^ T cells found in the negative controls (Nil tubes) were subtracted from those obtained in the experimental (Ag1 and Ag2) tubes to get the final adjusted AIM^+^ T-cell frequencies (thus possibly including negative adjusted values). An AIM-reactive status was defined when the relative frequencies of AIM^+^CD4^+^ and/or AIM^+^CD8^+^ T cells (i.e., percentages of total CD4^+^ or CD8^+^) from Ag1 and/or Ag2 tubes were greater than 0.05%. Samples from Mitogen and Nil tubes were also used as positive and negative controls for AIM analysis (**Fig 1B**). Data were analyzed using FlowJo software (Treestar).

### Statistical analysis

Numerical variables (e.g., antibody titers, IFN-γ levels, frequencies of virus-specific AIM^+^ T cells) were reported as mean values ± standard deviation or as mean with minimum and maximum values. Categorical variables (e.g., reactive and nonreactive status) were reported as counts or proportions. Since data were not normally distrubuted, as verified by Kolmogorov-Smirnov test, the following non-parametric statistical tests were used for analysis: Mann-Whitney U test for comparisons between unpaired numerical variables; Fisher’s exact test and Chi-square test for comparisons between categorical variables; Spearman’s rank test for correlations between two variables. A Spearman’s rho value >0.7 was considered as strong correlation, between 0.5 and 0.7 as moderate correlation, and <0.5 as weak correlation. Cohen’s kappa was used to assess the agreement between two assays. A Cohen’s kappa value >0.8 was considered as perfect agreement, between 0.6 and 0.8 as substantial agreement, between 0.4 and 0.6 as moderate agreement, between 0.2 and 0.4 as fair agreement, and <0.2 as slight agreement. Two-tailed *P* values <0.05 were considered significant. Statistical analysis and graphical representation of the data were performed using Graphpad Prism 8 software (La Jolla, US).

## Results

### T-cell responses after infection or vaccination

Among 22 unvaccinated cases, blood samples were QFN-reactive in 10 (45.5%) and AIM-reactive in 14 (63.6%). All QFN-reactive samples were also AIM-reactive, resulting in substantial agreement between the two assays (Cohen’s kappa: 0.65) (**Table 1**). However, AIM assay was more capable of detecting virus-specific responses, testing positive—in both CD4^+^ and CD8^+^ T-cell compartments—even in a case in which QFN was negative and both anti-S1 and anti-N antibodies were undetectable.

**Table 1:** Antibody and T-cell reactivity results: Concordance between assays and differences between cases (unvaccinated individuals with prior documented symptomatic infection) and controls (vaccinees with no prior symptomatic infection).

	UNVACCINATED CONVALESCENTS (CASES)			VACCINATED DONORS (CONTROLS)		
IMMUNOASSAY	Non-reactive subjects (n.)	Reactive subjects (n.)	Cohen’s kappa (degree of agreement)	Non-reactive subjects (n.)	Reactive subjects (n.)	*P* value (Fisher exact test)
Anti-S1 IgG	4	18		0	13	0.2735
Anti-S1 total antibodies	1	21		0	13	1
Anti-N antibodies	3	19		4	5	0.1504
QFN	12	10		1	12	**0.0099**
AIM	8	14		1	12	0.1094
Concordance (IgG vs. QFN)	4	10	0.3125 (fair agreement)	0	12	0.1001
Discordance (IgG vs. QFN)	0	8		0	1	1
Concordance (IgG vs. AIM)	2	12	0.1200 (slight agreement)	0	12	0.4831
Discordance (IgG vs. AIM)	2	6		0	1	1
Concordance (QFN vs. AIM)	8	10	**0.6452 (substantial agreement)**	0	11	**0.0116**
Discordance (QFN vs. AIM)	4	0		1	1	0.3333

S1, Spike protein subunit 1; N, Nucleocapsid protein; QFN, QuantiFERON; AIM, Activation-Induced Marker; vs., versus; n., numbers.

Among 13 vaccinated controls, both QFN and AIM assays were positive in 12 (92.3%), although the two assays were discordant in 2 subjects (**Table 1**).

**Fig 2:**
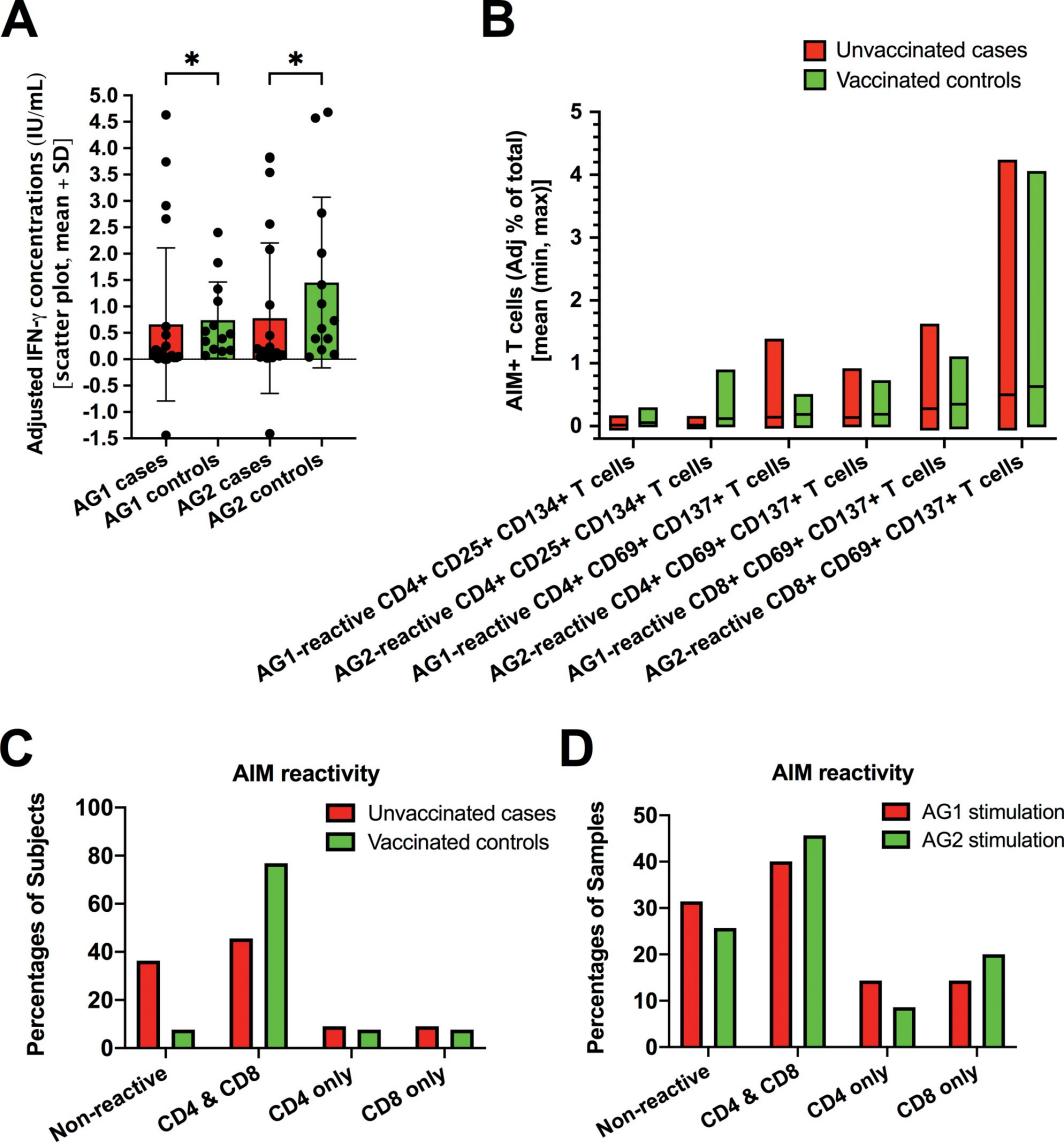
SARS-CoV-2-specific cellular immune responses in late convalescents and vaccinees. ***A)*** Differences between unvaccinated individuals with prior documented symptomatic infection (cases) and fully vaccinated asymptomatic donors (controls) in the adjusted concentrations of interferon-gamma (IFN-γ) released in supernatants following peripheral blood exposure to SARS-CoV-2-specific antigenic pools during incubation in the AG1 or AG2 tubes of QFN kit. Data are represented in scattered box plots and expressed as mean ± standard deviation (SD). *P < 0.05 (Mann-Whitney). IU/mL, international units per milliliter. ***B)*** Adjusted relative frequencies of AIM^+^ (CD25^+^ CD134^+^) CD4^+^, AIM^+^ (CD69^+^ CD137^+^) CD4^+^, and AIM^+^ (CD69^+^ CD137^+^) CD8^+^ T cells obtained in cases or controls upon AG1 or AG2 peptide stimulation. Data are represented in box plots showing the mean plus minimum and maximum values. ***C)*** Proportions of subjects, among cases or controls, showing either AIM reactivity in CD4^+^ and/or CD8^+^ T-cell compartments or nonreactivity. ***D)*** Proportions of samples (cases + controls) showing either AIM reactivity in CD4^+^ and/or CD8^+^ T-cell compartments or nonreactivity, upon AG1 or AG2 stimulation.

Proportions of QFN-negative results were significantly higher in cases compared to controls (**Table 1**). Consistently, mean IFN-γ concentrations in supernatants were lower in the former than in the latter (**Fig 2A**). On the other hand, in QFN-reactive samples, IFN-γ levels did not differ between cases and controls, following either Ag1 or Ag2 stimulation (not shown).

No statistical difference emerged in AIM reactivity between cases and controls, with similar frequencies of AIM^+^CD4^+^ and/or AIM^+^CD8^+^ T cells observed upon Ag1 or Ag2 stimulation (**Fig 2B**).

In cases as in controls, AIM reactivity was most often detected concomitantly in the CD4^+^ and CD8^+^ T-cell compartments (**Fig 2C**). Although Ag1 stimulation was supposed to be specific for CD4^+^ T cells according to the manufacturer, we actually found that Ag1 also activated CD8^+^ T cells, in agreement with other authors’ observations [20], possibly due to further processing of peptides by circulating antigen-presenting cells [20] or to the expansion of unconventional MHC-II-restricted CD8^+^ T-cell clones [21, 22]. In fact, AIM^+^ T cells were most often detected concomitantly in CD4^+^ and CD8^+^ compartments for both Ag1 and Ag2 stimulations, even though Ag1 stimulation resulted in non-significantly higher proportions of AIM^+^ T cells in the CD4^+^ compartment only compared to Ag2 stimulation (**Fig 2D**).

In both cases and controls, the great majority of AIM^+^CD8^+^ T cells showed the phenotype of terminally differentiated effector memory CD45RA^+^ T cells (T_EMRA_, CCR7^-^CD45RA^+^, mean frequencies: 85% and 87%, respectively) (**Fig 1A**), exception done for one unvaccinated case in which effector memory cells were the most (T_EM_, CCR7^-^CD45RA^-^: 90%) and one vaccinated control in which central memory cells were instead preponderant (T_CM_, CCR7^+^CD45RA^-^: 72%). Specifically in these last two subjects, AIM^+^CD8^+^ T cells were > 4% of total CD8^+^, and the unvaccinated case was the only one that documented a recent reinfection (confirmed by swab), thus likely accounting for newly expanded and less chronically stimulated effector memory T-cell clones.

### Antibody responses after infection or vaccination

In unvaccinated cases, anti-S1 IgG were observed in 18 (81.8%), total anti-S1 antibodies (including IgA) were detected in 21 (95.5%), and anti-N antibodies were found in 19 (86.4%).

In vaccinated controls, total or IgG class anti-S1 antibodies were detected in all 13 subjects (100%), whereas anti-N antibodies (measured in 9 subjects) were detected in a lower proportion (55.6%).

**Fig 3:**
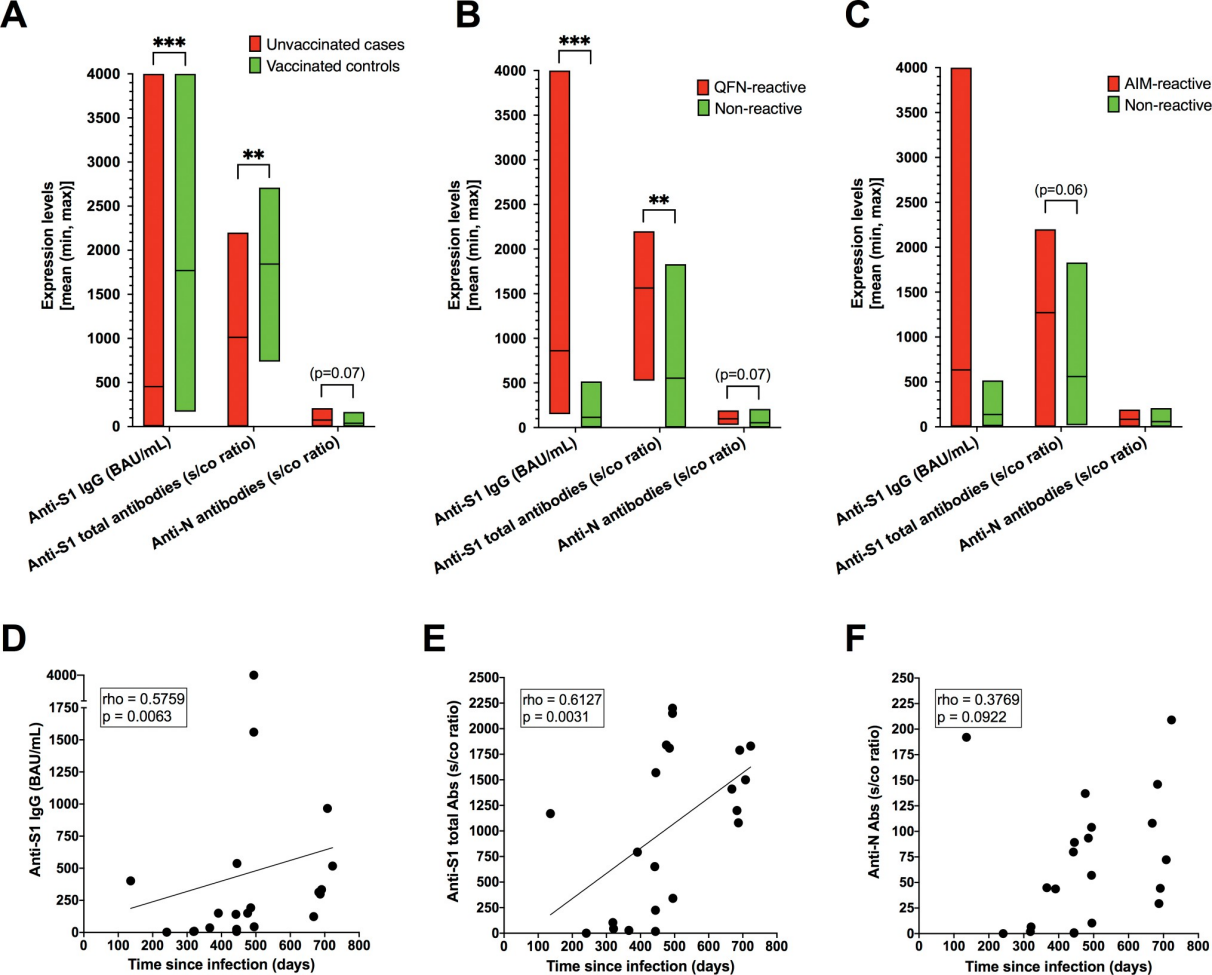
SARS-CoV-2-specific humoral immune responses in late convalescents and vaccinees. ***A)*** Differences in anti-S1 and anti-N antibody titers between convalescents (unvaccinated cases) and vaccinees (vaccinated controls). ***B)*** Differences in anti-S1 and anti-N antibody titers between QFN-reactive and QFN-nonreactive convalescents. ***C)*** Differences in anti-S1 and anti-N antibody titers between AIM-reactive and AIM-nonreactive convalescents. Data are represented in box plots showing the mean plus minimum and maximum values. ***P < 0.001; **P < 0.01 (Mann-Whitney). ***D-F)*** Spearman’s correlations between anti-S1 or anti-N antibody levels and time since prior infection in convalescents. BAU/mL, binding antibody units per milliliter; s/co ratio, sample to cut-off ratio.

No significant differences were observed between cases and controls in positive or negative serological test results (**Table 1**). Nevertheless, anti-S1 antibody titers were significantly higher in vaccinated controls, whereas anti-N antibody titers tended to be higher in unvaccinated cases (**Fig 3A**).

QFN-reactive unvaccinated cases showed significantly higher levels of circulating antibodies than QFN-nonreactive cases (**Fig 3B**), whereas numerical—not statistical—differences in antibody levels were observed between AIM-reactive and AIM-nonreactive cases (**Fig 3C**).

In our small case series of unvaccinated individuals, antibody levels were observed to increase with time since previous infection (**Fig 3D–3F**).

### Coordination between cellular and humoral responses in naturally acquired immunity

In unvaccinated individuals with prior infection, we found signs of coordination between T-cell (cellular) and antibody (humoral) responses to SARS-CoV-2 antigens, and between CD4^+^ and CD8^+^ T-cell compartments.

**Fig 4:**
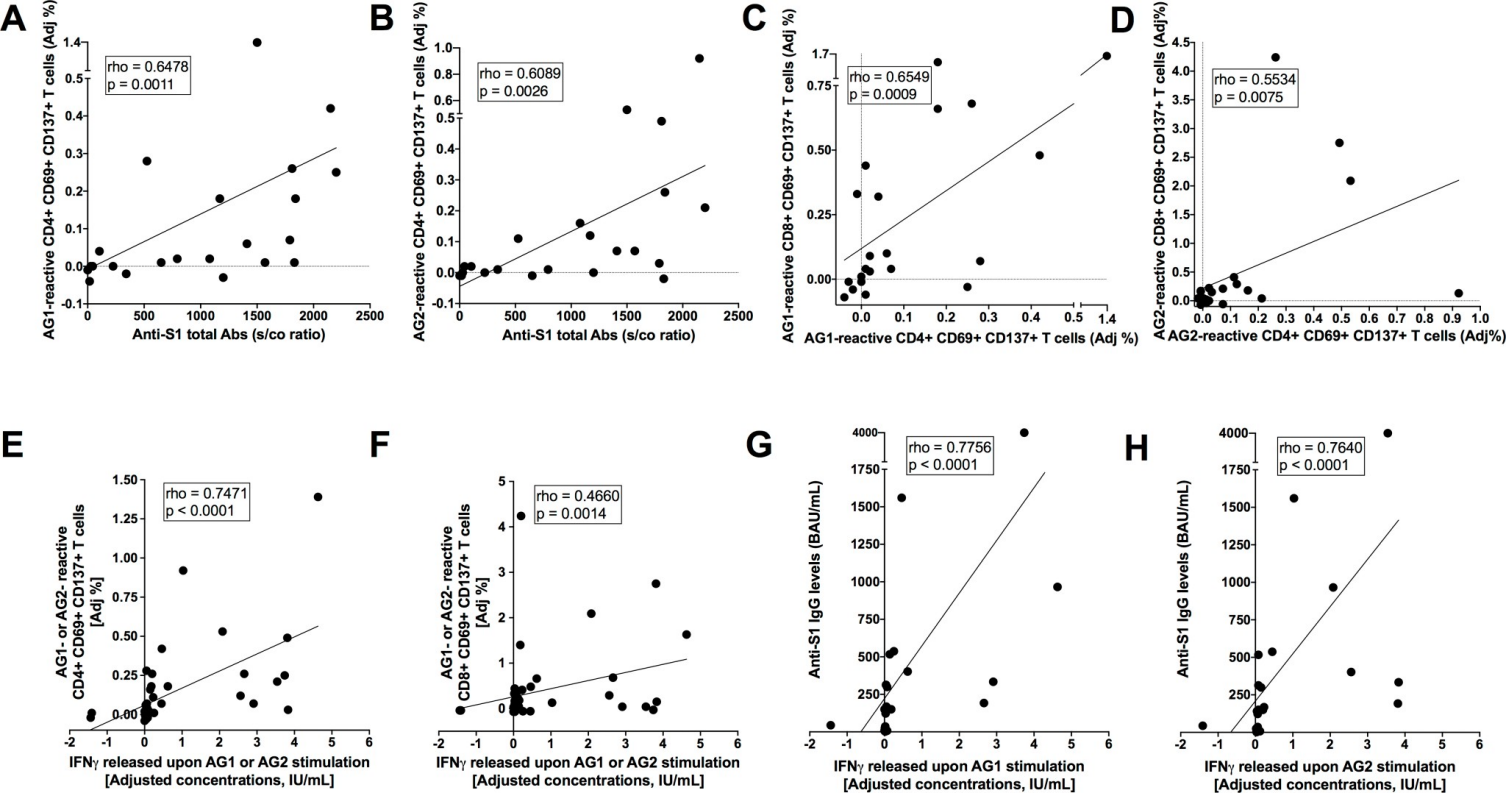
Coordinated adaptive immune responses against SARS-CoV-2 in late convalescents. ***A-B)*** Spearman’s correlations between AIM^+^ (CD69^+^ CD137^+^) CD4^+^ T-cell frequencies and anti-S1 antibody levels upon AG1 or AG2 peptide stimulation. ***C-D)*** Spearman’s correlations between AIM^+^ (CD69^+^ CD137^+^) CD4^+^ and AIM^+^ (CD69^+^ CD137^+^) CD8^+^ T-cell frequencies upon AG1 or AG2 peptide stimulation. ***E-F)*** Spearman’s correlations of AIM^+^ (CD69^+^ CD137^+^) CD4^+^ or AIM^+^ (CD69^+^ CD137^+^) CD8^+^ T-cell frequencies with concentrations of interferon-gamma (IFN-γ) released upon AG1 or AG2 stimulation. ***G-H)*** Spearman’s correlations of anti-S1 IgG levels with concentrations of IFN-γ released upon AG1 or AG2 stimulation. Adj %, adjusted percentages; IU/mL, international units per milliliter; BAU/mL, binding antibody units per milliliter.

In fact, frequencies of S-reactive AIM^+^ (CD69^+^CD137^+^) CD4^+^ T cells significantly correlated with anti-S1 antibody levels (**Fig 4A and 4B**). Anti-S1 and anti-N antibody titers correlated with each other, such that S1-specific (Ag1-reactive) AIM^+^CD4^+^ T-cell frequencies were also correlated with anti-N antibody levels (not shown). Furthermore, frequencies of AIM^+^CD4^+^ T cells correlated with those of AIM^+^CD8^+^ T cells (**Fig 4C and 4D**). Correlations were in general slightly stronger after Ag1 rather than Ag2 stimulation (**Fig 4A–4D**).

Higher concentrations of IFN-γ released during QFN assays correlated strongly with increased levels of AIM^+^CD4^+^ T cells and anti-S1 IgG, and more weakly with higher frequencies of AIM^+^CD8^+^ T cells (**Fig 4E–4H**).

### Differences between reactive and nonreactive status in unvaccinated individuals with prior infection

**Fig 5:**
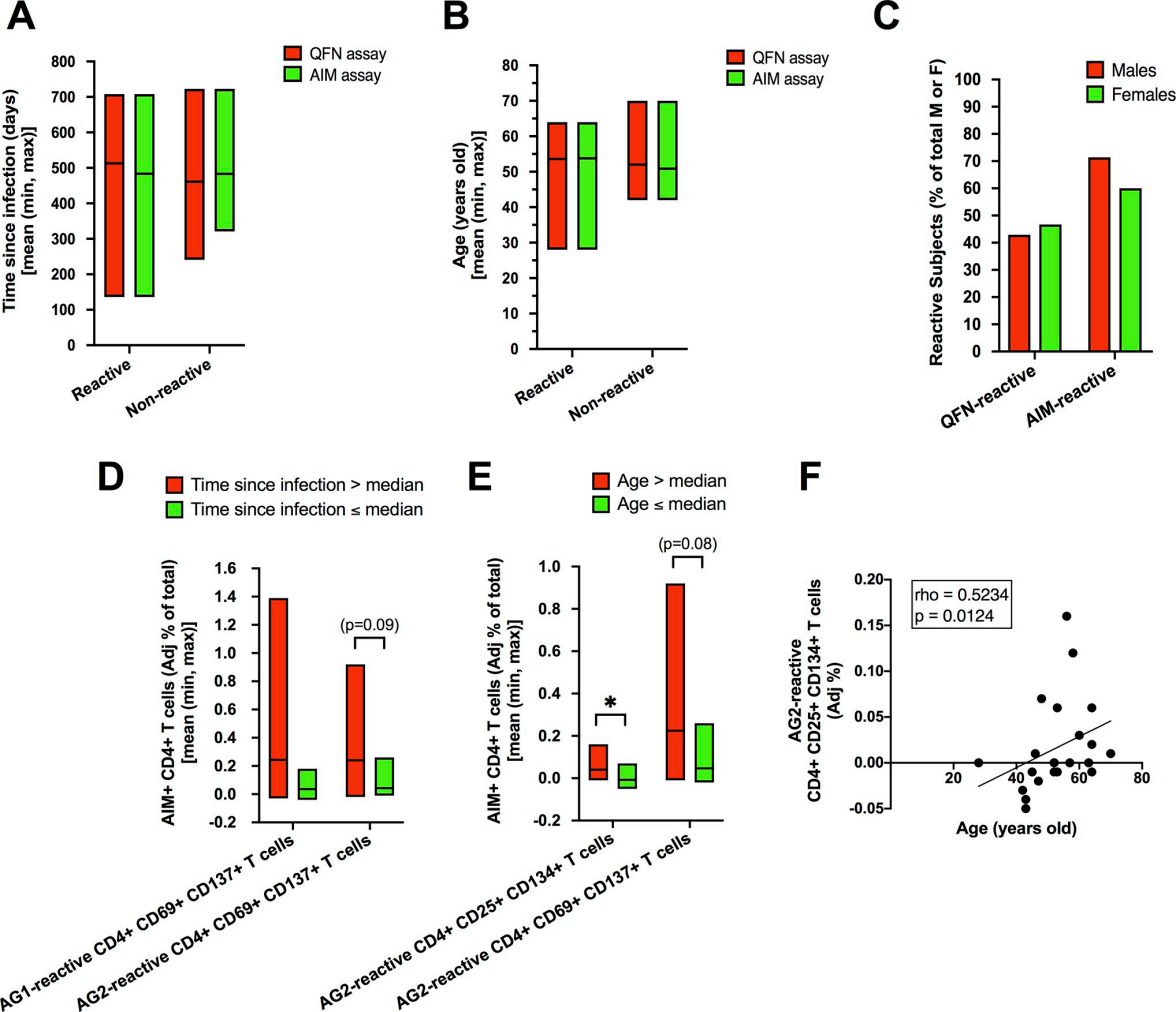
Differences between reactive and nonreactive convalescents and associations of T-cell reactivity with time since infection and age. ***A-B)*** Mean time since infection and mean age in reactive or nonreactive convalescents, as assessed by QFN or AIM assays. ***C)*** Proportions of male and female convalescents with QFN-reactive or AIM-reactive status. ***D)*** Differences in adjusted relative frequencies of AIM^+^ (CD69^+^ CD137^+^) CD4^+^ T cells between convalescents with longer or shorter time since infection. ***E)*** Differences in adjusted relative frequencies of AIM^+^ (CD25^+^ CD134^+^) CD4^+^ or AIM^+^ (CD69^+^ CD137^+^) CD4^+^ T cells between older and younger convalescents. Data are represented in box plots showing the mean plus minimum and maximum values. *P < 0.05 (Mann-Whitney). ***F)*** Spearman’s correlation between adjusted relative frequencies of AIM^+^ (CD25^+^ CD134^+^) CD4^+^ T cells and age.

Among unvaccinated convalescents, no differences in time since previous infection, age, or gender were overall observed between QFN-reactive or AIM-reactive and nonreactive ones (**Fig 5A–5C**).

However, at the level of single T-cell subsets, as mentioned above, the subject reporting the highest frequencies of AIM^+^CD8^+^ T cells (up to 4.24% after Ag2 stimulation) was the one reporting a recent reinfection (documented by swab) 20 days before the study.

Furthermore, particularly after Ag2 stimulation, AIM^+^CD4^+^ T cells tended to be higher in subjects with longer time since previous infection and modestly increased with age (**Fig 5D and 5E**). More specifically, it was the frequencies of AIM^+^ (CD25^+^CD134^+^) CD4^+^ T cells that correlated significantly with age (**Fig 5F**).

## Discussion

This study highlights that immunocompetent individuals with prior SARS-CoV-2 infection develop and maintain in the medium-long term cellular and humoral memory responses. Rates of T-cell reactivity and antibody positivity, as assessed by AIM and serological assays, do not significantly differ from those observed in vaccinees, confirming and extending recent findings of equally high T-cell responses following SARS-CoV-2 infection and COVID-19 vaccination [23].

Upon blood cell exposure to S-derived epitopes, we report that previously infected subjects display coordinated memory responses across all branches of adaptive immunity. Significant correlations were found between the expansion of AIM^+^CD4^+^ and AIM^+^CD8^+^ T cells, and between AIM^+^CD4^+^ T-cell frequencies and serum antibody levels. Indeed, coordination is an essential prerequisite for efficient antiviral responses [2, 6], as effectively occurred in our series of individuals with predominantly mild disease or with a favorable final outcome.

Moreover, we underscore that the magnitude of both cellular and humoral S-specific responses is closely associated with the amounts of IFN-γ released, thus confirming a primary role for the T_H_1 signature in orchestrating a protective and long-lasting antiviral response [2, 5, 6, 13, 17].

This study further extends, up to 2 years, our previous evidence of protection given by naturally acquired immunity [10, 11]. Indeed, our present data suggest that some memory responses may even increase with time since infection, in accordance with other authors’ observations [6, 24].

Furthermore, we highlight that convalescents with a previously documented symptomatic infection show circulating anti-N antibodies more frequently and at higher titers, whereas asymptomatic vaccinees show a substantially polarized anti-S response with minor anti-N responses [8], the latters probably reflecting previous exposure to cross-reactive endemic coronaviruses [25, 26] or, alternatively, hybrid immunity with silent infection [27]. In either case, stronger anti-N responses may help attenuate the pathogenicity of emerging VOCs with highly mutated S protein [2, 12, 28].

On the other hand, we found a lower rate of QFN reactivity, corresponding to lower mean levels of IFN-γ released, and lower anti-S1 antibody titers, in unvaccinated convalescents compared to vaccinees. Nevertheless, in QFN-reactive unvaccinated cases, IFN-γ production was not *per se* impaired, while, in some QFN-nonreactive cases, we found evidence of IFN-γ-independent AIM reactivity, not strictly linked to high antibody titers. In fact, AIM assay was able to detect virus-reactive subjects that were QFN-nonreactive and even antibody-negative, since it detects virus-specific T-cell reactivity regardless of cytokine response, thereby including both T_H_1-type and non-T_H_1-type responses. That implies the presence of at least 3 subsets of late convalescents based on immunological memory and T-cell commitment, namely: T_H_1-reactive (QFN-positive, AIM-positive, with higher antibody titers) (in our case series, 46%); non-T_H_1-reactive (QFN-negative, AIM-positive, with variable antibody titers) (18%); and pauci-reactive (QFN-negative, AIM-negative, with lower antibody titers) (36%). Therefore, combining QFN and AIM assays may increase overall detection of SARS-CoV-2-experienced individuals and allow for their stratification according to different grades of protection against reinfection or relapse, which may require different monitoring and vaccination strategies.

Whereas the vast majority of vaccinees (regardless of whether they are only vaccinated or hybrid) shows a memory response polarized on IFN-γ production and circulating IgG class antibodies (T_H_1-reactive), part of the naturally immunized subjects may instead display different T-cell commitments associated with other cytokines and antibody classes (non-T_H_1-reactive), based on individual background and severity of previous COVID-19 disease [13, 29, 30]. In particular, virus-specific AIM^+^CD4^+^ T cells derived from patients with mild disease may express not only IFN-γ but also IL-10 and TGF-β, thus promoting immunoregulatory Tr1 or Treg responses against hyperinflammation [2, 28–32] and IgA switching against mucosal reinfection [33], while other non-T_H_1 protective responses may be driven by virus-specific T_FH_ cells [4], thus fostering affinity maturation of neutralizing antibodies, even against VOCs [34]. Furthermore, memory T cells from convalescents who experienced moderate disease may express IL-9 and IL-4 (self-limiting T_H_9 responses), while those with severe disease highly express GM-CSF and IL-6 (T_H_17 or “T_H_-GM” responses), and those with critical disease highly express IL-6 and IL-10 (T_H_2 responses) [30–32].

Improved diagnostic accuracy given by the combination of QFN with AIM (CD69, CD137, CD25, CD134) was previously seen in tuberculosis [35–37], and looks promising in COVID-19 as well. Various combinations of markers have been used for AIM assays in COVID-19 [6, 7, 12, 13, 23, 24]; in agreement with others [38], the most of AIM^+^CD4^+^ T cells and the most of statistical correlations were herein found using CD69 and CD137. Multiparametric flow cytometry enables to easily study rare and heterogeneous virus-reactive T-cell populations [38]. Our in-house defined combination may be more consistent, less time-consuming and less expensive than combining other techniques, since it is based on common peptide stimulation for both assays and avoids more complex procedures such as ELISpot [5, 26], FluoroSpot [12], Luminex [23], or T-cell costimulation with anti-CD28/CD49d monoclonal antibodies and ICS [5, 15, 20]. Wakui and colleagues recently found a correlation between QFN and AIM results in mRNA vaccinees [19]. Here, we report a substantial agreement between QFN and AIM assays in late convalescents, reflecting the occurrence of T_H_1-type responses in roughly half of unvaccinated cases, with significant correlations found between IFN-γ concentrations and AIM^+^ T-cell frequencies.

In both convalescents and vaccinees, we detected signs of immunosenescence, such as the expansion of T_EMRA_ in CD8^+^ [5, 12, 28, 30] and the increased expansion of AIM^+^ (CD25^+^CD134^+^) CD4^+^ T cells with age. It will be interesting to elucidate possible relations of these phenotypes with the expansion of CD28^null^ T cells, as seen in other viral infections [39] and inflammatory disorders [40–42]. Similarly to T_EMRA_, in fact, CD28^null^ T cells are long-lived and repeatedly stimulated effector memory cells, which can be activated by alternative costimulation exerted by CD134 and CD137 [40, 41], and show high IFN-γ production [39, 42, 43]. Of note, SARS-CoV-2-specific CD8^+^ T_EMRA_ up-regulate MHC-II genes [43], which might at least in part account for bidirectional activation of T cells following incubation with CD4^+^ T-cell epitopes (e.g., Ag1 stimulation), as reported here and elsewhere [20].

This study has several limitations, including small sample size, sample heterogeneity in terms of time since infection and epidemiologically prevalent viral strain over time, different median time since immunization between convalescents and vaccinees, and absence of serial samples. On the other hand, in all subjects, at least 4 months had elapsed since immunization, thus a suitable time to assess antiviral memory T-cell responses, which are substantially preserved across variants; moreover, it was interindividual variability that ultimately allowed us, in this preliminary cross-sectional study, to detect statistical associations between variables.

In conclusion, albeit with a limited sample, our study attempts to shed further light on the adaptive immune memory of late convalescents, highlighting similarities and differences compared to vaccinees. Measuring cellular immune responses may importantly integrate antibody tests as correlates of protection and we suggest that AIM assay represents a valuable complement to QFN assay for the detection and stratification of SARS-CoV-2-experienced individuals. Assessing virus-specific T-cell reactivity quantitatively (e.g., minimum protective AIM^+^ T-cell frequencies), qualitatively (e.g., QFN^+^/T_H_1 *vs*. QFN^-^/non-T_H_1 responses), and temporally (e.g., persistence of long-lived T-cell phenotypes) will be key to optimizing public health strategies in the next future.
